# Treatment of Actinomycosis1Read before the Nebraska State Veterinary Association.

**Published:** 1896-06

**Authors:** V. Schaefer

**Affiliations:** Tekamah, Neb.


### TREATMENT OF ACTINOMYCOSIS.[1]

By DR. V. SCHAEFER,
TEKAMAH, NEB.

As we have actinomycosis in two forms, that is, one form affecting the glandular and soft structures about the head, neck, and sometimes the sternum and other parts of the body, and the other, which affects the bones and teeth of the upper and lower jaw, I will describe my treatment separately.

In the glandular form, or that in which the disease is manifest by soft tumors, I make an incision into the tumor large enough to admit one or two fingers, seldom larger; and aim to cut the centre as near as possible; evacuate the pus, if there be any, with a syringe; explore the cavity in the centre of the tumor with the finger, and break off all the granulation on the inside, as all these tumors are formed in the centre.

When I have found any actinomycotic cavity lined or filled with these granulations, as said before, I break them down with

[1] Read before the Nebraska State Veterinary Association.

my finger or a blunt instrument and use a six-inch curved scissors to remove everything which is loose, thus enlarging the cavity as much as possible. In a little oakum wrap from one drachm to one ounce of chloride of zinc, according to the size of the tumor or cavity. Insert this into each cavity as far up as possible. In very large cavities it is best to divide the chloride of zinc into several portions in order that it may come in contact with all parts of the cavity. Fill the remaining cavity with oakum and stitch it to retain the packing. Treat every tumor or abscess that may be found on the animal in like manner.

With a hypodermic syringe inject from one to two ounces of tincture of iodine, diluted with double the amount of alcohol, around and into the tumor and in the maxillary space. The quantity to be injected is to be governed by the size of the animal, using the most in large animals; where the tumors are large and deep-seated push the needle well down to the base so as to get the iodine deep into the tissue. In from ten to fourteen days the tumor will slough out and leave a healthy granulating sore, which will heal in the course of four or five weeks from the time of operation, depending somewhat on the stage of suppuration.

We also meet with some cases where there seems to be no localization of the disease, only appearing as a large soft swelling of all the lower parts of the jaw and throat, and sometimes an emaciated condition of the animal. In still other cases the tongue is so swollen that it protrudes, causing inability to eat or drink. In these cases cast the animal, and with a hypodermic syringe inject from one to two ounces of tincture of iodine diluted with alcohol into all parts of the swelling, and particularly in the maxillary space, which is the favorite seat for my injections.

Let the disease exist where it may, wherever there is much swelling I push my needle, which is a strong and rather short one, full length into the swelling. I have only treated a few of the last-named cases, but the treatment was satisfactory, the animal recovering in each case. In some cases a second operation was necessary where new abscesses formed. I treated two especially bad cases where several operations were necessary and where I tested the iodine in the treatment of actinomycosis, and since adopted the present method. In the spring of 1890 I removed a tumor about the size of a man's head from the brisket of a steer; another as large as a double fist from the maxillary space just below and a little to the anterior part of the larynx; and a smaller one from the anterior part of the head below the ear. I cut out the tumors until I thought there were no parts of them left, having used only a small amount of chloride of zinc and no iodine, thinking I had nothing but healthy wounds to treat. In a few days I operated on a similar case in a steer, but without the tumor at the sternum. In about six weeks I was again called to operate on the first steer. To my surprise I found that all the tumors had returned and grown as large as when first examined and the animal not in good condition, having lost flesh. I decided not to use a knife this time, as there was no abscess in the tumors, but instead a fungus or hyperplastic tissue. I wanted to test the iodine injection alone, so I cast the animal and injected the iodine as described before. Soon after the owner reported the animal was doing better and was gaining in flesh. In five or six weeks I cast him again and made injections. This time I discovered a large abscess on the side of the neck. I opened it, washed it thoroughly with water, and injected iodine in and around the abscess. In catching the steer we drove him on a stock scale and thus had an opportunity to weigh him. This the owner did each time. From the first to the second iodine injection the animal gained one hundred pounds, and the tumors were not more than half as large as at the first injection, and were quite healthy-looking. In six weeks I made a third injection, and this time found another abscess on the opposite side of the neck. By this time the animal had gained one hundred and thirty pounds. The tumors were almost entirely gone; the animal was fat, and his hair glossy-looking, and in five or six weeks the animal was sold in the Omaha market without any objections. The second steer proved the same as the first; the growths returning as large as before, but looking worse. I made two iodine injections, and a complete recovery was the result.

These were the last cases in which I removed tumors or enlargements, but since have operated on more than two hundred and fifty animals, and nearly everyone recovered. The operation just described is a simple one, and more than 95 per cent. will recover.

The form affecting the lower jaws is not so easily nor so successfully treated as that affecting the soft structures. It depends greatly upon what portion of the jaw is affected and at what stage treatment is attempted. We find cases at such advanced stages in which the jaw is increased many times its normal size, the bone softened, and the whole structure of the jaw a perforated mass of rarefied bone. In such cases it is useless to attempt treatment. Treatment of the upper jaw is not so successful as the lower one, which I am about to describe.

I operate when there is no external opening, and in most all cases where there is an opening, but where the jaw is not increased more than three or four times above normal, or in all cases where the enlargement in the jaw is circumscribed, and examination proves that there is enough comparatively sound bone to prevent the jaw from breaking after removal of the diseased portion; the case is more hopeful if the anterior portion of the ramus is affected than the posterior, especially if the disease extends back to the angles.

In operating on a case before suppuration has set in, and where there is only an enlargement of the jaw-bone without an external opening, extract the teeth in the enlarged part, and sound one tooth on each side, anterior and posterior, to make certain that all the diseased part will be included in the operation. When the greater part or the whole jaw is enlarged extract all the teeth. After extracting the teeth make an external opening with the trephine or bone-gouge through the inferior surfaces as if to punch out the teeth. Enlarge this opening anteriorly and posteriorly as desired in order to remove all the fangs or roots of the teeth that may exist. Remove all soft or diseased material between the inner and outer plates of the jaw. Make an oakum plug to fit the full length of the cavity in the jaw. Have an assistant hold it in place by twine looplet around it and near its ends, and pass the strings out through the hole in the jaw. Draw the plug down into the jaw, just a little to hold it in place and tight enough to prevent anything escaping into the mouth. Fill the remaining space with chloride of zinc and oakum, aiming to pack it completely from the outer opening. Lay a large pledget of oakum over the external opening and tie the strings which hold the plug on the inside over the pledget on the outside; the strings thus holding both inner and outer plugs in position. Inject iodine along the jaw and in the maxillary space, and let the animal up. The first few days it will not eat nor drink much, but it will soon begin to improve, and the jaw will get well in from three to six weeks, depending greatly at what stage the operation was performed. When the jaw has already an external opening with a running sore, with or without a fungus attached to its inferior surface, treat it in the same way, using the original opening, and in most cases where the disease is located in the jaw our attention is generally called in the last-named stage.

If there is enough solid bone in the jaw to allow removal of most of the diseased bone without danger of breaking it off, the animal usually recovers. Both lower and upper jaws are treated in the same manner, modified according to circumstances. In a number of very bad cases two or three operations were performed, there being so much of the bone affected as not to allow the removal of all the diseased portion at one time.

After removing the most diseased parts and treating as above stated, the jaw will become less in size and firmer, and the disease more circumscribed. In a few weeks another operation can be performed more satisfactorily, and recovery has nearly always followed. Some may ask, Why not treat with iodide of potassium, as this is said to give good results? But this treatment does not suit most of the Western feeders or owners of young cattle, requiring more work, attended to regularly, quarantined, and in many cases the animals are wild and almost unmanageable. I have advised feeders to try the iodide of potassium treatment, but was told they would rather pay for the operation than have the trouble themselves. I find it is about the only operation I perform which is appreciated by the owner and in which he feels repaid for his expense.

It gives me an extended practice and adds considerably to my income at the end of the year.

